# Health-related quality of life among general practice patients with differing chronic diseases in Germany: Cross sectional survey

**DOI:** 10.1186/1471-2458-8-246

**Published:** 2008-07-21

**Authors:** Hong-Mei Wang, Martin Beyer, Jochen Gensichen, Ferdinand M Gerlach

**Affiliations:** 1 Institute of Social Medicine and Family Medicine, School of Medicine, Zhejiang University, 388 Yuhang Tang Rd., 310058 Hangzhou, PR China; 2 Institute for General Practice, Johann Wolfgang Goethe University, Theodor-Stern-Kai 7, 60590 Frankfurt a. M., Germany; 3 Department of Family Medicine, Medical School, Friedrich-Schiller-University, Bachstr. 18, 07740 Jena, Germany

## Abstract

**Background:**

This study was carried out to compare the HRQoL of patients in general practice with differing chronic diseases with the HRQoL of patients without chronic conditions, to evaluate the HRQoL of general practice patients in Germany compared with the HRQoL of the general population, and to explore the influence of different chronic diseases on patients' HRQoL, independently of the effects of multiple confounding variables.

**Methods:**

A cross-sectional questionnaire survey including the SF-36, the EQ-5D and demographic questions was conducted in 20 general practices in Germany. 1009 consecutive patients aged 15–89 participated. The SF-36 scale scores of general practice patients with differing chronic diseases were compared with those of patients without chronic conditions. Differences in the SF-36 scale/summary scores and proportions in the EQ-5D dimensions between patients and the general population were analyzed. Independent effects of chronic conditions and demographic variables on the HRQoL were analyzed using multivariable linear regression and polynomial regression models.

**Results:**

The HRQoL for general practice patients with differing chronic diseases tended to show more physical than mental health impairments compared with the reference group of patients without. Patients in general practice in Germany had considerably lower SF-36 scores than the general population (*P *< 0.001 for all) and showed significantly higher proportions of problems in all EQ-5D dimensions except for the self-care dimension (*P *< 0.001 for all). The mean EQ VAS for general practice patients was lower than that for the general population (69.2 versus 77.4, *P *< 0.001). The HRQoL for general practice patients in Germany seemed to be more strongly affected by diseases like depression, back pain, OA of the knee, and cancer than by hypertension and diabetes.

**Conclusion:**

General practice patients with differing chronic diseases in Germany had impaired quality of life, especially in terms of physical health. The independent impacts on the HRQoL were different depending on the type of chronic disease. Findings from this study might help health professionals to concern more influential diseases in primary care from the patient's perspective.

## Background

Primary care addresses the most common problems in the community by providing preventive, curative, and rehabilitative services to maximize health and well-being [1]. "The Ecology of Medical Care Revisited," by Green et al. [2] provided a broader, still useful framework for thinking about the organization of health care, medical education, and research originally developed by White et al. [3]. The "ecology" implies that patients who are seen in primary care are a special population, differing both from the general population and patient populations in a secondary care setting. Health information related to patients outside primary care is not comparable to patients in this setting [4].

Health-related quality of life (HRQoL), representing people's subjective assessment of their sense of well-being and ability to perform social roles, has been well accepted as a health indicator in medical interventions or health surveys. The HRQoL of patients with one single chronic disease has been explored in primary care [5-7]. For example, the quality of life of patients with prolonged back pain was significantly worse than that of the average Finn [5]. The mean HRQoL score of patients with an untreated depression at baseline was quite low. Eight percent of patients rated their health state as worse than death [6]. In another study, newly diagnosed patients with Type 2 diabetes in general practice compared with screening-detected patients reported more diabetes-related symptom distress shortly after the diagnosis, and a consistently worse mental health status at 6 months and 12 months [7]. However, most studies on the HRQoL of patients with chronic diseases in the primary care setting are focused on one chronic disease in particular. Relatively little is known about HRQoL profiles for primary care patients with differing chronic diseases, and the differences in HRQoL for primary care patients in comparison to the general population [8]. Even if a substantial percentage of chronically ill patients suffers from more than one chronic condition, or multimorbidity in this setting [9,10], a small number of studies focused on the relationship between multimorbidity and HRQoL [11], or the combined effects of pairs of chronic diseases on the HRQoL [8,12]. Systematic investigations into the independent influence of the multiple chronic diseases in primary care are still limited.

In the current study, we compared the HRQoL of patients in general practice with differing chronic diseases with the HRQoL of patients without chronic conditions. We evaluated the HRQoL profiles of consecutive patients presenting for general practice consultation in Germany and compared them with those of the general population. Finally, we tried to estimate the effect of each chronic disease independently of the effects of multiple confounding variables.

## Methods

### Setting

A cross-sectional survey was conducted in 20 general practices from the regional mailing list of the Institute of General Practice, University Hospital Schleswig-Holstein between June and December 2002. Each practice was asked to complete 50 questionnaires. Several large practices were ready to include more patients.

### Study population

On given days chosen by each practice, all consecutive patients over 14 years old were invited to self-administer the survey before consultation. Each participant put the completed questionnaire into an anonymous envelope and gave it to his or her doctor. For each patient, the doctor recorded up to three diagnoses for the actual encounter and three existing chronic conditions or major health problems. The protocol was approved by University Hospital Schleswig-Holstein Ethics Committee and all subjects provided informed consent.

### Questionnaire

*The Medical Outcomes Study 36-item Short Form Health Survey (SF-36)*, consists of 36 items which measure eight scales: physical functioning (PF), role limitations due to physical problems (RP), bodily pain (BP), general health (GH), vitality (VT), social functioning (SF), role limitations due to emotional problems (RE), and mental health (MH). On the basis of these separate subscales, component summary scores can be calculated to provide a global measure of physical (Physical Component Summary score, PCS) and mental functioning (Mental Component Summary score, MCS), respectively. The scale scores range from 0 to 100, with higher scores indicating a better health status. The PCS and MCS have been standardized on the basis of a normative US general population data set, with the mean set at 50 (SD 10) [13].

*The EQ-5D questionnaire *is a simple generic HRQoL instrument. It comprises two pages: on the first page respondents record the extent of their problems in each of five dimensions (self-classifier) concerning mobility, self-care, usual activities, pain/discomfort, and anxiety/depression; on the second page, they record their perception of overall health on a visual analogue scale (EQ VAS). EQ VAS scores may range between 0 (worst imaginable health) and 100 (best imaginable health) [14].

Besides HRQoL instruments, demographic questions were included in the questionnaire. These variables included age, gender, marital status, employment status, smoking, educational attainment (high school or more/having a professional certificate), academic degree or qualification, medical insurance, and self-reported severe illness experience.

### Statistical methods

The diagnoses and morbidity data were coded according to the International Classification of Primary Care (ICPC-2) [15]. Thirteen chronic diseases were extracted from the codes: hypertension, diabetes, asthma or chronic obstructive pulmonary disease (COPD), heart disease of any kind, stroke, osteoarthritis (OA) of the knee, other joint diseases, depression, cancer, chronic back pain, polyarthritis, other mental illness, and migraine. Multimorbidity was defined as reporting two or more health problems of the selected thirteen chronic diseases.

The SF-36 scores were calculated according to the established scoring algorithms for the German version of the SF-36 [16]. The disease groups were partly overlapping because of multimorbidity, so it was not possible to compare across groups. Instead, unadjusted SF-36 scale scores of each disease group were compared with those of the reference group without any of the thirteen chronic conditions. The original response choices in the EQ-5D self-classifier (no problem, some/moderate problems, and extreme/unable to) were regrouped into two categories (reporting ***no ***problem and reporting ***any ***problem). As well, the proportions reporting ***any ***problem for each disease group were compared with those for the reference group by means of the chi-square test.

The eight SF-36 scale and two summary scores of patients in general practice were compared with the general population in Germany [17]. Both parametric *t *test and non-parametric Mann-Whitney *U *test were used to allow the comparison of results, allowing for the possibility that the data might not match normal distribution. The proportions reporting ***any ***problem in the EQ-5D dimensions for each disease group were compared with those for the general population [18] using the chi-square test. The mean EQ VAS for general practice patients was compared with those for the general population using the *t *test. The EQ index derived from the responses to the five dimensions was not analyzed in this paper, because we think the index is conventionalized and can be regarded as a societal valuation of the respondent's health state, not the respondent's own assessment of his or her health state.

The independent effects of differing chronic diseases, self-reported severe illness experience, age, gender, marital status, employment status, smoking, education attainment (high school or more/having a professional certificate), academic degree or qualification, and medical insurance on the PCS and MCS scores of the SF-36 and EQ VAS score were analyzed using multivariable linear regression (Variable selection procedure: Enter). Additional regression analyses explored the possibility of non-linear relationships between age as a continuous variable and the PCS and MCS scores of the SF-36 and EQ VAS score. We also examined interactions between age and chronic diseases.

All statistical analyses were carried out using SPSS for Windows (version 13.0; SPSS Inc., Chicago, IL, USA).

## Results

Of the 1231 patients recruited by 20 practices, 1041 agreed to participate, representing a response rate of 84.6%. Twenty-four questionnaires were deleted because diagnoses were missing (in one practice). Eight other questionnaires were completed by accompanying persons and therefore excluded.

Among the 1009 eligible respondents, 61.4% were women, and the mean age was 48.5 years (range 15–89, SD 17.4). A total of 428 (42.4%) respondents did not have any of the selected chronic diseases, 377(37.4%) had one, 155 (15.4%) had two, and 49 (4.9%) had three or more of the diseases. More than one third (35.1%) of patients with the selected chronic diseases had at least one other chronic disease. The 190 non-respondents had a mean age of 57.0 years (range 14–91, SD 18.6). A total of 176 non-respondents reported the reasons why they refused to participate. Thirty-two percent of them attributed their refusal to visual or comprehension problems. The demographic characteristics of the sample are shown in Table 1. Patients with chronic diseases were more likely than the reference group to be older, unemployed, and less educated.

**Table 1:** Demographic characteristics of general practice patients by disease groups

	Mean age (SD)	Female (%)	Now married (%)	Employed or self-employed (%)	Having high school or more education or a professional certificate (%)	Having an academic degree or qualification (%)	Statutory insurance (%)
Overall (n = 1009)	48.5 (17.4)	61.4	62.8	50.8	83.4	24.0	87.6
Reference group (n = 428)	40.5 (15.8)	65.2	58.6	59.6	87.3	23.2	86.9
Hypertension (n = 217)	61.5 (13.2)	54.1	68.5	35.5	77.9	26.5	86.8
Diabetes (n = 78)	63.0 (12.1)	47.1	73.5	29.9	70.6	21.7	88.2
Asthma/COPD (n = 54)	52.1 (18.5)	57.1	56.3	36.0	81.6	31.9	86.0
Heart disease (n = 78)	64.8 (15.7)	50.7	60.6	20.8	72.2	25.8	89.0
Stroke (n = 3)	55.5 (23.3)	66.7	66.7	0	50	0	100
OA knee (n = 17)	67.8 (8.0)	52.9	82.4	11.8	52.9	5.9	76.5
Other joint diseases (n = 30)	61.7 (14.6)	48.0	65.4	34.6	73.1	25.0	80.8
Depression (n = 63)	52.2 (17.6)	83.0	50.0	47.4	80.7	17.6	91.1
Cancer (n = 38)	58.8 (13.3)	72.4	71.9	35.5	78.1	19.4	96.9
Back pain (n = 193)	51.1 (15.2)	58.1	67.5	53.8	82.2	26.8	88.2
Polyarthritis (n = 13)	55.6 (15.3)	75.0	76.9	38.5	61.5	0	84.6
Other mental illness (n = 29)	43.4 (14.3)	65.2	34.8	60.9	82.6	36.4	91.3
Migraine (n = 25)	42.0 (12.1)	90.5	63.6	68.2	90.9	22.7	95.5

Table 2 compares the unadjusted SF-36 scale scores of patients with each chronic disease with those of the reference group. Stroke was removed from the subsequent analysis because only three patients were in this group. Overall, significant differences between all disease groups and the reference group existed except for other mental illness and migraine. Patients in each disease group had one or more lower SF-36 scale scores than those in the reference group. The SF, RE, and MH scale scores evaluating mental health were significantly lower only for patients with depression and other mental illness. In depression patients, to note, all eight scale scores were significantly lower.

**Table 2:** Mean SF-36 scale scores for general practice patients by disease groups

	PF	RP	BP	GH	VT	SF	RE	MH
Reference group (n = 428)	82.8	69.1	68.6	64.0	54.1	77.7	72.6	67.0
Hypertension^2 ^** (n = 217)	71.5***	58.4**	62.9**	56.3***	56.8	79.2	77.8	72.4*
Diabetes^2 ^** (n = 78)	66.5***	56.7*	62.4	58.0**	59.1*	80.4	80.4*	75.1**
Asthma/COPD^1 ^*** (n = 54)	68.1***	42.7***	59.5*	47.4***	47.7*	73.8	71.4	66.4
Heart disease^1 ^*** (n = 78)	59.7***	44.2***	57.2**	49.7***	46.9*	74.7	61.7	67.4
OA knee^1 ^*** (n = 17)	62.7**	46.7**	48.8**	53.0*	57.7	80.2	89.6*	72.6
Other joint diseases^1 ^* (n = 30)	63.4**	48.2*	47.4**	56.1	51.1	75.8	69.1	65.8
Depression^2 ^** (n = 63)	68.3***	48.9**	55.5**	54.0***	39.2***	59.0***	39.6***	46.6***
Cancer^1 ^* (n = 38)	66.6**	49.2	58.6	51.8**	52.0	71.3	63.6	63.5
Back pain^1 ^*** (n = 193)	72.2***	52.7***	49.7***	58.4**	51.8	76.5	70.9	67.2
Polyarthritis^1 ^* (n = 13)	57.8***	46.2*	46.8**	46.6*	47.9	70.2	55.6	63.3
Other mental illness^1 ^(n = 29)	80.9	61.0	69.0	54.1	47.0*	66.1	53.8*	56.9*
Migraine^1 ^(n = 25)	78.9	60.9	55.1*	60.5	49.2	75.5	84.1	68.7

PF: physical functioning; RP: role physical; BP: bodily pain; GH: general health; VT: vitality; SF: social functioning; RE: role emotional; MH: mental health. * *P *< 0.05, ** *P *< 0.01, *** *P *< 0.001 ^1. ^Multivariate Analysis of Variance (Hotelling T^2^) for groups with equal covariance matrices: there were significant differences between all these disease groups and the reference group except other mental illness and migraine groups. ^2. ^Mann-Whitney *U *test with Bonferroni correction for groups with unequal covariance matrices: there were significant differences between these disease groups and the reference group.

**Figure 1:**
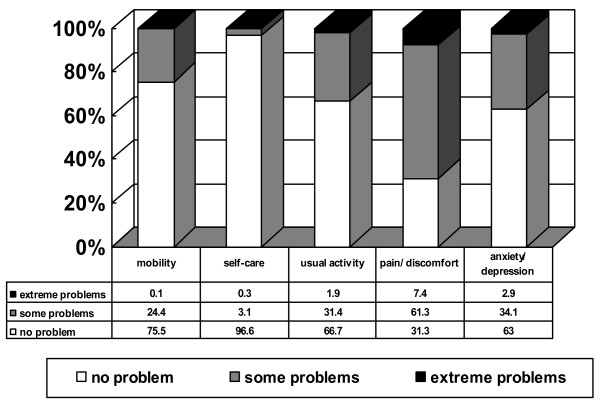
Distribution of the EQ-5D self-classified health states of patients in German general practice.

Complete data in all EQ-5D dimensions were only available for 914 respondents. A total of 707 (77.4%) of respondents reported problems in one or more of the EQ-5D dimensions. Pain/discomfort was the dimension most frequently (68.7%) noted as causing problems. (Figure 1). A total of 830 respondents gave answers in the EQ VAS score with a mean of 69.2 (range 0–100, SD 19.8). Table 3 compares the unadjusted proportions reporting ***any ***problems in the EQ-5D self-classifier for each disease group, with those for the reference group. The presence of any chronic disease tended to increase the risk of reporting ***any ***problem in at least one dimension. Similarly, the proportions reporting ***any ***problem in the dimension of anxiety/depression were significantly higher only for patients with depression and other mental illness.

**Table 3:** The likelihood of reporting ***any ***problem in the EQ-5D self-classifier by disease groups

	Number (proportion) of respondents reporting ***any ***problem				
	
	Mobility	Self-care	Usual activity	Pain/discomfort	Anxiety/depression
Reference group (n = 428)	83(20.0%)	10(2.4%)	120(29.0%)	248(60.2%)	149(35.8%)
Hypertension (n = 217)	49(25.5%)	7(3.6%)	48(25.4%)	134(70.2%)*	54(28.4%)
RR (95% CI)	1.27(0.94–1.73)	1.51(0.58–3.90)	0.88 (0.66–1.17)	1.17 (1.03–1.32)	0.79 (0.61–1.03)
Diabetes (n = 78)	24(34.8%)**	2(3.0%)	17(25.0%)	45(65.2%)	20(29.0%)
RR (95% CI)	1.74(1.19–2.53)	1.23(0.28–5.50)	0.86(0.56–1.34)	1.08(0.90–1.31)	0.81(0.55–1.20)
Asthma/COPD (n = 54)	18(36.0%)*	4(8.0%)	23(46.0%)*	39(78.0%)*	21(42.9%)
RR (95% CI)	1.80(1.18–2.72)	3.30(1.08–10.14)	1.59(1.13–2.22)	1.30 (1.10–1.53)	1.20(0.84–1.70)
Heart disease (n = 78)	21(31.3%)*	3(4.5%)	25(37.9%)	49(75.4%)*	20(29.9%)
RR (95% CI)	1.56(1.04–2.34)	1.85(0.52–6.55)	1.31(0.93–1.84)	1.25(1.07–1.47)	0.83(0.56–1.23)
OA knee (n = 17)	9(56.3%)**	1(6.3%)	6(37.5%)	16(94.1%)**	6(35.3%)
RR(95% CI)	2.81(1.75–4.50)	2.58(0.35–18.96)	1.29(0.68–2.48)	1.56(1.36–1.80)	0.98(0.51–1.90)
Other joint diseases (n = 30)	9(36.0%)	2(8.3%)	9(37.5%)	21(84.0%)*	9(36.0%)
RR (95% CI)	1.80(1.03–3.13)	3.44(0.80–14.84)	1.29(0.76–2.22)	1.40(1.16–1.68)	1.00(0.59–1.72)
Depression (n = 63)	17(30.4%)	1(1.8%)	26(47.3%)**	41(75.9%)*	45(83.3%)***
RR (95% CI)	1.51(0.97–2.35)	0.74(0.10–5.65)	1.63(1.19–2.24)	1.26(1.06–1.49)	2.33(1.95–2.77)
Cancer (n = 38)	11(31.4%)	0(0%)	19(54.3%)**	26(74.3%)	16(47.1%)
RR (95% CI)	1.57(0.93–2.65)	- (-)	1.87(1.33–2.63)	1.23(1.00–1.52)	1.31(0.90–1.92)
Back pain (n = 193)	50(28.4%)*	8(4.6%)	73(41.2%)**	146(83.4%)***	61(34.7%)
RR (95% CI)	1.42(1.05–1.92)	1.90(0.76–4.73)	1.42(1.13–1.79)	1.39(1.25–1.54)	0.97(0.76–1.23)
Polyarthritis (n = 13)	4(30.8%)	1(7.7%)	5(38.5%)	13(100%)**	7(53.8%)
RR (95% CI)	1.54(0.66–3.55)	3.18 (0.44–23.01)	1.33 (0.66–2.68)	1.66 (1.54–1.80)	1.50 (0.89–2.53)
Other mental illness (n = 29)	3(11.1%)	1(3.6%)	13(48.1%)*	20(71.4%)	17(63.0%)**
RR(95% CI)	0.55(0.19–1.64)	1.48(0.20–11.12)	1.66(1.09–2.53)	1.19(0.93–1.52)	1.76(1.28–2.41)
Migraine (n = 25)	7(29.2%)	0(0%)	5(20.8%)	20(83.3%)*	6(25.0%)
RR (95% CI)	1.46(0.76–2.79)	-(-)	0.72(0.32–1.59)	1.38(1.14–1.68)	0.70(0.34–1.41)

* *P *< 0.05, ** *P *< 0.01, *** *P *< 0.001 by chi-square tests on the difference in the proportion of respondents reporting ***any ***problem between patients with the selected chronic diseases and the reference group, df = 1. RR = the relative risk of reporting ***any ***problem of respondents with the selected chronic diseases compared with the reference group.

**Figure 2:**
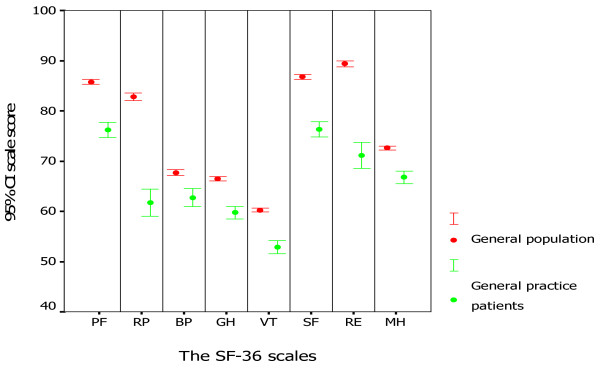
The SF-36 scale scores for patients in general practice and those for the general population in Germany.

The comparison of the SF-36 scores for general practice patients and for the general population is shown in Table 4, Figure 2. Both parametric and non-parametric methods gave virtually identical results. Patients in general practice had a significantly lower score in each scale or summary score (*P *< 0.001 for all). The differences observed in the scales concerning daily role limitations (RP and RE) were particularly salient. Table 5 shows the difference in the proportion of respondents reporting ***any ***problem in the EQ-5D self-classifier between general practice patients and the general population. The proportions of general practice patients reporting ***any ***problems were significantly higher (*P *< 0.001 for all) than those of the general population in all dimensions except for the self-care dimension. The mean EQ VAS for general practice patients was lower than that for the general population (69.2 versus 77.4, *P *< 0.001).

**Table 4:** Comparison of the SF-36 scale and summary scores of general practice patients and those of the general population in Germany [17]

	Mean (SD)	*t*^1^	P	Median (IR)	*Z*^2^	P
PF						
General practice patients	76.2(25.0)			85.0(34.7)		
General population	85.8(20.4)	11.56	<0.001	95.0(20.0)	-13.94	<0.001
RP						
General practice patients	61.7(41.9)			75.0(75.0)		
General population	82.8(32.4)	14.74	<0.001	100(25.0)	-16.93	<0.001
BP						
General practice patients	62.7(28.8)			62.0(59.0)		
General population	67.7(25.9)	5.11	<0.001	72.0(49.0)	-4.56	<0.001
GH						
General practice patients	59.7(19.1)			62.0(27.0)		
General population	66.5(18.2)	10.32	<0.001	67.0(27.0)	-10.42	<0.001
VT						
General practice patients	52.9(20.0)			55.0(30.0)		
General population	60.3(17.8)	10.85	<0.001	60.0(25.0)	-10.73	<0.001
SF						
General practice patients	76.3(24.5)			87.5(37.5)		
General population	86.8(19.6)	12.81	<0.001	100(25.0)	-14.38	<0.001
RE						
General practice patients	71.1(40.2)			100(66.7)		
General population	89.4(26.5)	13.42	<0.001	100(0)	-16.81	<0.001
MH						
General practice patients	66.8(19.4)			68.0(31.2)		
General population	72.6(16.6)	8.90	<0.001	76.0(20.0)	-8.64	<0.001
PCS						
General practice patients	45.2(11.1)			47.3(16.7)		
General population	48.5(9.3)	8.43	<0.001	51.0(10.9)	-8.82	<0.001
MCS						
General practice patients	46.9(11.8)			49.8(17.2)		
General population	51.0(8.7)	9.82	<0.001	53.0(9.6)	-8.79	<0.001

PF: physical functioning; RP: role physical; BP: bodily pain; GH: general health; VT: vitality; SF: social functioning; RE: role emotional; MH: mental health; PCS: physical component summary score; MCS: mental component summary score.

^1.^*t *test. ^2. ^Mann-Whitney *U *test.

**Table 5:** Percentage of patients and the general population with problems in the EQ-5D self-classifier [18]

	General practice patients			General population
		
	Moderate problems	Extreme problems	Moderate/extreme problems	Moderate/extreme problems
Mobility	24.4	0.1	24.5	17***
Self-care	3.1	0.3	3.4	3
Usual activity	31.4	1.9	33.3	10***
Pain/discomfort	61.3	7.4	68.7	28***
Anxiety/depression	34.1	2.9	37.0	4***

* *P *< 0.05, ** *P *< 0.01, *** *P *< 0.001 by chi-square tests on the difference in the proportion of respondents reporting ***any ***problem between general practice patients and the general population, df = 1.

Multivariable linear regression showed that OA of the knee, back pain, experience of own illness, old age, having never married, and seeking work were significant predictors of a lower SF-36 PCS score. Depression, experience of own illness and divorced/separated were significant risk factors, and back pain was a significant mitigating factor in the regression equation for the SF-36 MCS score. Depression, cancer, experience of own illness, and having never married were significant risk predictors of the EQ VAS score, while patients with more education tended to have a higher score (Table 6). The residuals from the linear regression models approximated a normal distribution.

**Table 6:** Multivariable regression models for the SF-36 summary scores and the EQ VAS score versus chronic diseases and demographic characteristics

Variable	PCS^a^		MCS^b^		EQ VAS^c^	
			
	β^d^(SE)^e^	*P *value	β^d^(SE)^e^	*P *value	β^d^(SE)^e^	*P *value
*Chronic diseases*						
Hypertension	-1.131(1.457)	0.438	2.613(1.696)	0.124	1.279(2.871)	0.656
Diabetes	-1.282(2.297)	0.577	5.236(2.674)	0.051	6.196(4.523)	0.172
Asthma/COPD	-4.299(2.370)	0.070	-0.394(2.758)	0.886	-8.833(4.997)	0.078
Heart disease	-2.994(2.193)	0.173	-0.135(2.553)	0.958	-2.077(4.492)	0.644
OA knee	-11.009(4.092)	0.007	8.918(4.763)	0.062	0.315(9.433)	0.973
Other joint diseases	-5.339(3.339)	0.111	-0.260(3.886)	0.947	-8.303(7.007)	0.237
Depression	-1.255(2.197)	0.568	-8.664(2.557)	0.001	-11.222(4.460)	0.012
Cancer	-1.912(2.994)	0.523	-0.936(3.485)	0.788	-16.312(5.845)	0.006
Back pain	-5.126(1.332)	<0.001	3.322(1.550)	0.033	0.089(2.688)	0.974
Polyarthritis	-6.960(4.046)	0.086	5.458(4.710)	0.247	-7.721(8.817)	0.382
Other mental illness	2.586(3.297)	0.433	-6.999(3.838)	0.069	-6.841(6.174)	0.269
Migraine	-2.616(3.120)	0.402	1.364(3.631)	0.707	4.457(6.830)	0.514
						
*Demographic characteristics*						
Self-illness	-3.001(1.120)	0.008	-5.566(1.304)	<0.001	-6.913(2.256)	0.002
Illness-family members	-1.568(1.166)	0.179	1.545(1.357)	0.255	1.098(2.351)	0.641
Gender	0.654(1.124)	0.561	-1.545(1.308)	0.238	-2.450(2.223)	0.271
Age	-0.118(0.057)	0.039	0.065(0.067)	0.328	-0.004(0.114)	0.973
High school or more education or a professional certificate	2.153(1.608)	0.181	2.254(1.872)	0.229	6.737(3.337)	0.044
Academic degree or qualification	0.654(1.184)	0.581	-0.251(1.378)	0.856	-1.449(2.389)	0.544

*Marital status (now married as reference)*						
Never married	-3.923(1.455)	0.007	-1.603(1.693)	0.345	-5.557(2.805)	0.048
Widowed	-4.316(2.395)	0.072	2.474(2.787)	0.375	-7.208(4.972)	0.148
Divorced/separated	0.403(2.046)	0.844	-7.951(2.382)	0.001	-5.603(4.062)	0.169

*Smoke(no smoker as reference)*						
Smoker	-1.632(1.224)	0.183	-0.518(1.425)	0.716	-3.372(2.468)	0.173
Ex-smoker	-1.249(1.206)	0.301	-0.966(1.403)	0.492	-3.357(2.407)	0.164

*Employment (employed or self-employed as reference)*						
Retired	-0.531(2.004)	0.791	0.021(2.333)	0.993	-6.745(3.881)	0.083
Keeping house	-2.283(1.649)	0.167	-0.065(1.919)	0.973	0.552(3.359)	0.869
Student	1.699(1.927)	0.378	-1.632(2.242)	0.467	2.514(3.798)	0.508
Seeking work	-6.156(2.686)	0.022	0.218(3.127)	0.945	-9.265(5.160)	0.073
Other	3.958(9.594)	0.680	3.817(11.167)	0.733	4.144(18.964)	0.827

*Medical insurance (statutory medical insurance as reference)*						
Commercial medical insurance	0.005(1.464)	0.997	1.996(1.704)	0.242	0.943(2.965)	0.751
Other	2.402(3.243)	0.459	0.181(3.774)	0.962	-4.325(6.417)	0.501

*Constant*	56.343(3.229)	<0.001	43.542(3.759)	<0.001	74.474(6.480)	<0.001

*F *value	4.292	<0.001	3.247	<0.001	2.565	<0.001

*R-square*	0.256		0.207		0.175	

^a ^PCS: physical component summary score.

^b ^MCS: mental component summary score.

^c ^EQ VAS: EQ-5D visual analogue scale.

^d ^Regression coefficient (linear relationship).

^e ^SE = standard error.

Table 7 shows the results of polynomial regression analyses using age in years, age squared, and age cubed. The non-linear association between age and the MCS and EQ VAS score were statistically significant (*P *< 0.05). The MCS score tends to decrease during the initial period of getting old, and then begins to increase. The EQ VAS score tends to have the inverse order. The relationships between age and the PCS score were considered to be linear association. The joint effects on the SF-36 PCS score between age and hypertension were less negative due to the positive interaction effect (*P *< 0.05). Interaction effects between age and all other chronic diseases were not statistically significant.

**Table 7:** Regression models for the SF-36 summary scores and the EQ VAS score using the age polynomials

Variables	PCS^a^		MCS^b^		EQ VAS^c^	
			
	β^d^(SE)^e^	*P *value	β^d^(SE)^e^	*P *value	β^d^(SE)^e^	*P *value
Age in years	-0.219(0.022)	<0.001	-1.210(0.503)	0.016	0.514(0.232)	0.027
Age squared			0.031(0.011)	0.005	-0.007(0.002)	0.006
Age cubed			<0.001 (<0.001)	0.003		
*Constant*	55.459(1.091)	<0.001	57.971(7.124)	<0.001	61.367(5.216)	<0.001
*F *value	99.931	<0.001	12.104	<0.001	7.421	0.001
*R-square*	0.110		0.043		0.019	

^a ^PCS: physical component summary score.

^b ^MCS: mental component summary score.

^c ^EQ VAS: EQ-5D visual analogue scale.

^d ^Regression coefficient (linear or non-linear relationship).

^e ^SE = standard error.

## Discussion

In this study, we found that general practice patients with chronic diseases seemed to have an impaired quality of life, compared with patients without any of the selected chronic conditions in a consecutive sample in Germany. Except for psychiatric disorders, these impairments were greater in physical functioning than in mental health. We also found that general practice patients in Germany had considerably lower scale scores and component summary scores for the SF-36 and higher proportions reporting ***any ***problems in all EQ-5D dimensions except for the self-care dimension, compared with the general population. Finally when we controlled for demographic variables such as age, gender, marital status, employment status, smoking, educational attainment, academic degree or qualification, medical insurance, and self-reported severe illness experience, we found that OA of the knee and back pain predicted of a lower SF-36 PCS score, depression predicted of a lower SF-36 MCS score, and that depression and cancer were risk factors for the EQ VAS score.

Twenty percent of consecutive patients visiting the general practice had more than one of the thirteen chronic diseases. The proportion of multimorbidity (two or more medical conditions) in general practice patients varied in previous studies: e.g. 3.6% [19], 16.2% [4] and 29.7% [9] of the population under study, which can be explained by the narrow, medium, or broad nosological spectrum respectively. Studies included only patients visiting the general practice during the study period might report a higher overall prevalence of multimorbidity than those where all patients registered in general practice were concerned [9]. It is difficult to compare these studies because of many differences in methodology, target population, and the number and type of diseases under study.

Based on the results of univariate analysis, patients with chronic diseases seemed to have a lower quality of life, compared with patients without any of the thirteen chronic conditions. Except for psychiatric disorders, these impairments were, as expected, stronger for dimensions of physical health than for dimensions of mental health. Patients with depression experienced both physical and mental health impairments significantly. Patients with hypertension and diabetes experienced better mental health than the reference group. Similar results were described in other studies [4,20-22]. However, some of the observed effects might reflect the combined result of the disease, comorbid conditions and demographics. In the multivariable regression models of our data, depression significantly impaired the SF-36 MCS score, while back pain affected mental health positively and other chronic diseases were "neutral". Rijken et al. assessed the separate and joint effects of cardiovascular disease, cancer, arthritis, chronic respiratory disease, diabetes mellitus, and thyroid dysfunction. They found physical functioning appeared to be impaired in all six diagnostic groups, whereas mental functioning was more or less comparable with reference data for the general population [12]. Increased multimorbidity was also found to be more strongly associated with a greater deterioration in physical functioning than in mental health in two other studies [23,24].

Quality of life rating is subjective and relative to a person's life expectations [25]. Patients suffering from chronic disease over a long period of time may become used to their illness. An important mediator of this psychological adaptation process is "response shift", which involves changing internal standards, values and the conceptualization of HRQoL [26,27]. Patients of advanced age and with chronic diseases might downscale their expectations for life and feel contented as long as they could stabilize their condition and be free from complications. As our results showed, old patients had better mental health, and ageing patients suffering from hypertension report less physical disability than could be expected from the separate effects of these two influential factors. Even though many chronic diseases often result in life-threatening complications and reduce life expectancy, these adverse events are typically preceded by many "silent" years. Asymptomatic status may be less likely to affect quality of life.

The clinically important difference (CID) reflects the amount of change (either improvement or decline) in HRQoL that is meaningful to patients and/or their health care providers [28]. The CID estimates for the patient-reported outcome measures depend on the rater and assessment methodology and may potentially vary for different questionnaires, different population and context. Published research indicates that a difference of 2.5–5 points in SF-36 PCS or MCS scores and 5–10 points in SF-36 scale scores among rheumatoid arthritis patients is considered clinically important [29,30]. The CIDs for the SF-36 scales established by the heart disease expert panel were generally greater than the CIDs agreed on by the asthma and COPD panels, and were all greater than the CID thresholds developed among patients with rheumatoid arthritis [31]. However, there is some evidence of commonality in the variety of approaches. For example, the estimates of the CID for the SF-6D and EQ-5D appear to be proportionally equivalent in the context of the range of utility scores for each scale [32]. In most circumstances, half a SD may serve as a default value for important patient-perceived change on HRQoL measures used with chronic disease patients in the absence of more specific information [33]. In the present study, the differences observed in the SF-36 scales concerning daily role limitations (RP and RE) and social functioning (SF) between general practice patients and the general population exceeded the value of 0.5 SD. This might help general practitioner initially evaluate and treat their patients.

The HRQoL for patients was measured with generic HRQoL measurements, the SF-36 and EQ-5D. Both the SF-36 and EQ-5D are applicable to a wide range of health conditions, which are suitable for the primary care setting. The SF-36 measures health status over the past four weeks and the EQ-5D measures health status on the day. The advantages of the SF-36 include its broader coverage of HRQoL dimensions (health profiles) [13], while the advantages of the EQ-5D include its brevity and simplicity, comparability across different populations, as well as its applicability in health-economic evaluations (preference-based measures) [14]. Correlations between SF-36 subscale scores and EQ dimensions were reported to be no more than 0.50 (considered to be a high correlation by Cohen [34]), except for the correlations between bodily pain (SF-36) and pain/discomfort (EQ-5D) (0.57) and mental health (SF-36) and anxiety/depression (EQ-5D) (0.50) [35]. Because of the limited number of responses (3) in each of the EQ dimensions (5), a ceiling effect may occur when measuring health among a relatively healthy population [18,35]. We expected the measurement of HRQoL using both the SF-36 and EQ-5D to present more meaningful information than only one of the forms alone, due to their different measurement attributes. Our investigation showed that both instruments reported impairments in comparable scales or dimensions for patients with any chronic disease, compared with the reference group. Neither of them reported impairments in facets of mental status for patients with any chronic disease except for patients with depression and other mental illness. Even though the two metrics are both intended to capture HRQoL, they seem to capture different aspects of it. For example, OA of the knee and back pain were significant predictors of lower SF-36 PCS but none of these were significant predictors of the EQ VAS score, while depression and cancer were significant predictors of a lower EQ VAS score but only depression had a negative effect on the SF-36 MCS. Compared with the general population, patients in general practice had a significantly lower score in each scale or summary score of the SF-36, while no difference was found in the proportion of respondents reporting ***any ***problem in the self-care dimension of the EQ-5D.

The associations between demographic variables and the HRQol were generally in line with literature. Self-reported severe illness experience is predictive for worse HRQoL. Old age is indicative of worse physical health and better mental health [18,20,35,36]. Patients not living with a partner reported worse HRQoL [18,20,35,36]. Educational attainment was associated with better HRQol [18,20,36], and unemployment was associated with poorer levels of HRQol [18,24,37]. Experience of illness of a family member had adverse effects on HRQoL [38,39], which was not observed in our study. Our study had a cross-sectional design, so it was not possible to determine causal relationships. Further longitudinal studies in the primary care setting will be required to test for the presence of associations between these demographics and the HRQoL and to fully interpret their clinical significance.

### Limitations of the study

The study reported here is part of a project to make a cross-cultural comparison of HRQoL for patients consulting in general practice between Germany and China. The study included more than 1000 patients. We could only use a convenience sample of general practices, but as the observation unit was the patient, we were confident that neither the regional limits nor the specificity of the study practices would substantially constrain the sample. The practices were basically comparable to the region. Of course, our survey is not easily comparable to other countries, as the "ecology" of primary care differs in every health care system.

There is difference between the HRQoL for general practice population and those for "stable" patients in the community. It is more salient for some diseases than others. For example, patients with back pain as their reason for encounter were likely to have worse pain and role limitations, whereas patients with hypertension as their reason for encounter saw the doctor probably for regular prescription and check. There is a bias with consecutive visit-based sampling: practice population has very different contact frequencies and thus more frequent users (likely to be sicker patients) are selected which will bias the associations in the direction of their own profile [40]. Our study might not generalize to the low users of general practice. Each practice was asked to complete 50 questionnaires. We would suppose they needed only two or three days to collect the questionnaires. Therefore the risk that one patient was questioned more than once seems negligible. However, we could not assume that there was no clustering of patient by practice. It remains a limitation. Home visit patients were not included. Non-respondents seemed to be older, which might lead to underestimation of the prevalence of morbidity and consequently influence the HRQoL of this population.

The number of patients with specific diseases such as polyarthritis was insufficient, which led to difficulties in showing a statistical significance for smaller effects. This suggests the possibility of false negative results (type II error). We performed a large number of hypothesis tests, which suggests the possibility of false positive results (type I error). The absence or presence of chronic diseases in our research sample was recorded by the doctors, which could preclude the possibility of confounding due to personal or mood characteristics [8]. However, we did not assess the severity of each condition, which may explain why the multivariable models did not account for a larger proportion of the variability in HRQoL scores (R-square 17.5 to 25.6% for linear regression models). Fortin et al. used the Cumulative Illness Rating Scale (CIRS) as a measure of disease severity in a primary care context, and revealed a stronger association with the HRQoL [23].

## Conclusion

This study indicated that general practice patients with differing chronic diseases in Germany had impaired quality of life compared with patients without chronic conditions and the general population, especially in terms of physical health. We found that OA of the knee and back pain predicted of a lower SF-36 PCS score, depression predicted of a lower SF-36 MCS score, and that depression and cancer were risk factors for the EQ VAS score. Findings from this study might help health professionals to concern more influential diseases in primary care from the patient's perspective. Further research is needed to identify the clinically important differences in HRQoL scores for primary care patients with differing chronic diseases. This research should also be done in patient samples representative of both high and low users of general practice, so that results may generalize to the broader practice population. Independent influences of both disease type and severity on the HRQoL need to be observed. Additionally, to determine causal relationships, further longitudinal studies in the primary care setting will be needed to test for the associations between the demographics and the HRQoL.

## Competing interests

The authors declare that they have no competing interests.

## Authors' contributions

HMW participated in the design of the study, carried out the study, performed the statistical analysis and drafted the manuscript. MB participated in the design of the study, supervised the data collection and entry and helped to draft the manuscript. JG and FMG participated in the design of the study and revised the manuscript. All authors read and approved the final manuscript.

## Pre-publication history

The pre-publication history for this paper can be accessed here:


